# Case report: Durable complete response of a mucosal melanoma of the rectum after neoadjuvant immunotherapy with ipilimumab plus nivolumab

**DOI:** 10.3389/fimmu.2024.1369190

**Published:** 2024-05-14

**Authors:** Oskar Krueger, Robin Eisenburger, Alpaslan Tasdogan, Lisa Zimmer, Elisabeth Livingstone, Eva Hadaschik, Sarah Theurer, Berthold Brodin, Dirk Schadendorf, Selma Ugurel

**Affiliations:** ^1^Department of Dermatology, University Hospital Essen, and German Cancer Consortium (DKTK), Partner Site Essen/Düsseldorf, Essen, Germany; ^2^Institute of Pathology, University Hospital Essen, University Duisburg-Essen, Essen, Germany; ^3^Gastropraxis, Neuss, Germany

**Keywords:** mucosal melanoma, immunotherapy, complete response, checkpoint inhibitors, neoadjuvant

## Abstract

Melanoma causes the majority of skin cancer-related deaths. Despite novel therapy options, metastatic melanoma still has a poor prognosis. Immune checkpoint inhibition (ICI) therapy has been shown to prolong overall survival in patients with advanced melanoma, but mucosal melanomas respond less favorably compared to melanomas of cutaneous origin. We report on a patient with a mucosal melanoma of the rectum diagnosed in June 2020. Since a surgical intervention in order to achieve a tumor-free situation would have required an amputation of the rectum, a neo-adjuvant systemic immunotherapy with ipilimumab and nivolumab was initiated. As restaging and colonoscopy after four doses of this combination immunotherapy showed a partial response, the patient decided against the pre-planned surgery and a maintenance therapy with nivolumab was started. Repeated colonoscopy showed a complete response after four doses of nivolumab. After ongoing ICI therapy with nivolumab and no evidence of tumor relapse, immunotherapy was stopped in July 2022 after nearly 2 years of continuous treatment. The patient remained tumor-free during further follow-up. Neo-adjuvant immunotherapy is getting more explored in advanced melanoma. By administering ICI therapy before surgical resection of an essentially operable tumor, a stronger and more diverse immunological response is supposed to be achieved. Our reported case demonstrates that this approach could also be effective in mucosal melanoma despite of its generally lower response to immunotherapy.

## 1 Introduction

The neoadjuvant use of immune checkpoint inhibitor (ICI) therapy in resectable skin cancers, particularly melanoma, is a major subject of current research. By administering ICI therapy before surgical resection of an essentially operable tumor, a stronger and more diverse immunological response is supposed to be achieved. Blank and coworkers showed favorable response and survival outcomes in stage III cutaneous melanoma patients treated with the CTLA-4 inhibitor ipilimumab plus the PD-1 inhibitor nivolumab in a neoadjuvant versus an adjuvant setting (1). Patel et al. demonstrated that a higher proportion of patients with resectable stage III/IV cutaneous melanoma achieved a 2-year event-free survival with combined neoadjuvant and subsequent adjuvant therapy with the PD-1 inhibitor pembrolizumab, compared to patients receiving only adjuvant pembrolizumab (2). A similar perioperative treatment regimen has been successfully tested using nivolumab plus the LAG-3 inhibitor relatlimab (3).

However, the efficacy of neoadjuvant ICI therapy in patients with mucosal melanoma are not as well studied. We here report a complete and durable response of an intestinal mucosal melanoma of the rectum upon neoadjuvant treatment with ipilimumab plus nivolumab.

## 2 Case presentation

**Figure 1:**
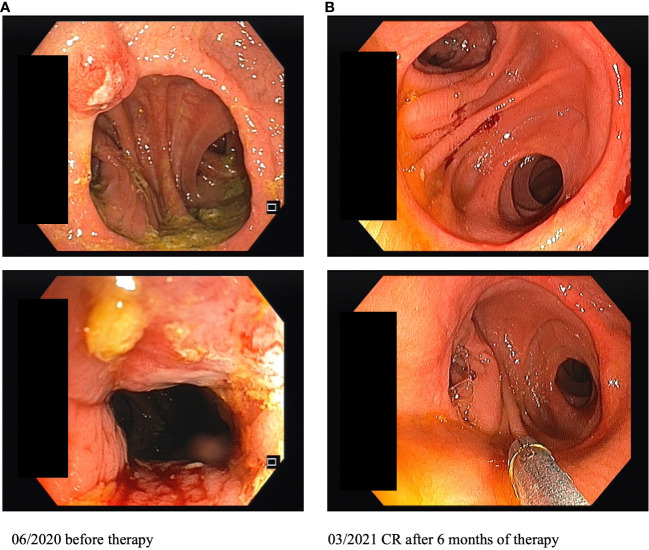
Images of colonoscopy on site of the previous rectocolostomy before and after 6 months of immunotherapy (CR, complete response).

**Figure 2:**
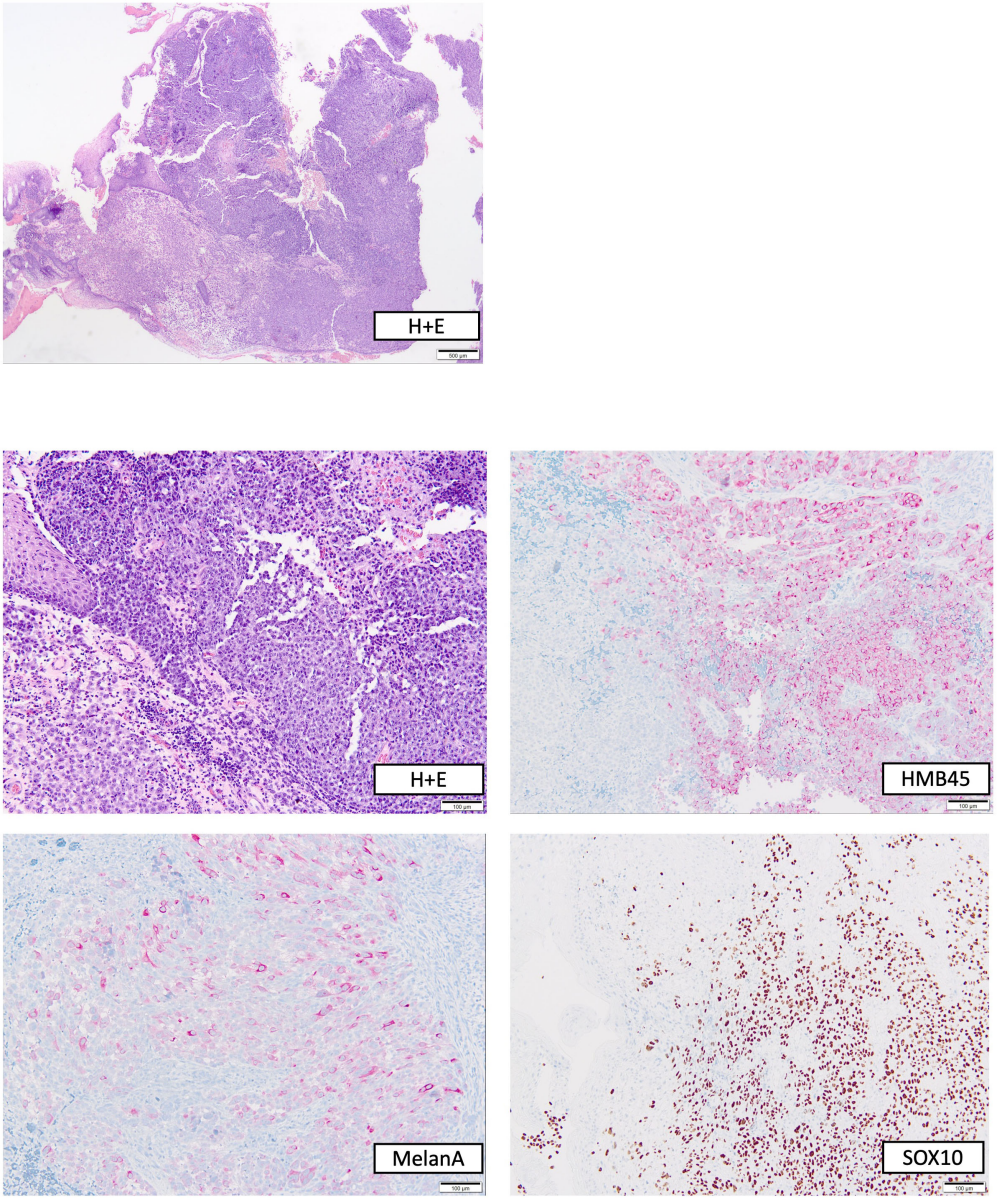
Histopathological work-up of the polypoid rectal tumor biopsied before start of immunotherapy. Top, overview stained with hematoxylin + eosin (H+E); bottom, H+E and immunohistochemical stainings in higher magnification.

During follow-up care for rectal cancer of a 76-year-old male patient diagnosed in January 2015 and treated with partial rectum amputation and FOLFOX chemotherapy, a new non-pigmented tumor lesion of 15 mm diameter was discovered localized on site of the previous rectocolostomy in June 2020 by colonoscopy (**Figure 1A**). As a recurrence of rectal cancer was primarily suspected, the coarse, polypoid tumor was biopsied. Histological work-up revealed mucosal melanoma with immunohistochemical positivity of S100, HMB45, melan A and SOX10 (**Figure 2**). PD-L1 expression was negative (0% of tumor cells stained). Molecular pathology showed no mutations in NRAS, NF-1 and KIT, but in BRAF (V600G) and SF3B1 (R625C). Disease staging by CT, MRI, and PET-MRI scan showed no evidence of metastases. The dermatological examination revealed no primary tumor on the skin or the visible mucosa. Serum LDH and S100B levels were within normal range.

The case was discussed in our interdisciplinary tumor board. Since a surgical intervention in order to achieve a tumor-free situation in this preoperated area would have required an amputation of the rectum, a neoadjuvant systemic immunotherapy was recommended. After shared decision making, we initiated a neoadjuvant ICI combination therapy with ipilimumab (3 mg/kg) plus nivolumab (1 mg/kg) Q3W in August 2020 with the aim to improve surgical options and hereby maintain the patient’s bowel continence.

Restaging and colonoscopy after four doses of ipilimumab plus nivolumab showed a partial response with a marked shrinkage of the polypoid tumor. Apart from immune-related thyroiditis and hypophysitis, which were both treated by hormone substitution (L-thyroxine, hydrocortisone), treatment was well tolerated. There was no delay in treatment, but the patient is still dependent on oral substitution therapy. Since at that time a significant partial response was already achieved, the patient decided against the surgery which was originally planned in the neoadjuvant approach. Thus, a maintenance therapy with nivolumab (480 mg Q4W) was started in November 2020. In March 2021, after four doses of nivolumab, repeated colonoscopy showed a complete response with total remission of the previously polypoid tumor lesion (**Figure 1B**). After ongoing ICI therapy with nivolumab and no evidence of tumor relapse, immunotherapy was stopped in July 2022 after nearly 2 years of continuous treatment. The patient is undergoing regular follow-up care including CT/MRI restaging as well as repeated colonoscopies. Until October 2023 the patient remained tumor-free. Written informed consent was obtained from the patient for the publication of this Case Report.

## 3 Discussion

To date, neoadjuvant therapy regimens have not been approved by the EMA or FDA for skin tumors in general, but are being investigated with great interest in clinical trials (4, 5). The combination regimen of ipilimumab plus nivolumab showed promising results in the neoadjuvant treatment of melanoma (1), so that its use is currently being compared with adjuvant nivolumab monotherapy in a clinical phase 3 trial in stage III cutaneous or unknown primary melanoma patients (NCT04949113).

The present case of a durable complete remission of a mucosal melanoma of the rectum upon neoadjuvant immunotherapy serves as a prime example for the immediate use of immune checkpoint blockade in cases of difficult operability, and the decision to forego primary tumor resection. Surprisingly, the mucosal melanoma of the patient exhibited a BRAF mutation, which would have made a neoadjuvant targeted therapy also a potential option for treatment (6). However, a pooled analysis of six clinical trials in patients with resectable stage III cutaneous malignant melanoma, tumor response was more durable after neoadjuvant immunotherapy, in particular with the combination of ipilimumab plus nivolumab, than with neoadjuvant BRAF/MEK inhibition (7). With the introduction of triple therapy regimens (combined BRAF/MEK inhibition plus PD-1 blockade) in the metastatic setting of cutaneous melanoma (8), trials using triple therapy in the neoadjuvant setting for BRAF-mutant melanoma may be seen in the future (9).

Mucosal melanomas in general respond less favorably to immunotherapy compared to cutaneous melanomas (10, 11). Therefore, it is surprising that the patient reported here responded so well to the neoadjuvant immunotherapy. However, study findings signal further use of neoadjuvant strategy with immune checkpoint inhibitor therapy in mucosal melanoma (11). In the end, the patient decided against a final surgical intervention to remove any residual tumor after clinical complete response, and thus ultimately received a definitive immunotherapy. Despite the rejection of the surgery a histological confirmation using a sample biopsy would have been useful in order to distinguish between complete and partial remission. However, the patient declined a biopsy due to concerns about anastomotic leakage.

This is a weakness of the neoadjuvant therapy approach, as patients can ultimately object to surgical therapy and therefore do not receive a neoadjuvant therapy concept as previously discussed, but rather definitive immunotherapy. As a result, baseline biomarkers such as tumor mutational status, tumor infiltrating lymphocytes and PD-L1 expression are also not available to correlate to outcome.

In the event of tumor progression, we would have switched the systemic therapy to a targeted therapy with combined BRAF and MEK inhibition as the mucosal melanoma of the patient exhibited a BRAF mutation.

There is compelling study data suggesting the switch from an adjuvant to a neoadjuvant approach in resectable stage III/IV melanoma. Further studies will be necessary to clarify whether a neoadjuvant versus adjuvant versus perioperative approach should be preferred dependent on relapse-free and overall survival outcomes of the patients.

## Data availability statement

The original contributions presented in the study are included in the article/supplementary material. Further inquiries can be directed to the corresponding author.

## Ethics statement

Ethical approval was not required for the studies involving humans because there was informed consent from the patient and it was just a case report. The studies were conducted in accordance with the local legislation and institutional requirements. The participants provided their written informed consent to participate in this study. Written informed consent was obtained from the individual(s) for the publication of any potentially identifiable images or data included in this article.

## Author contributions

OK: Writing – original draft. RE: Writing – review & editing. AT: Writing – review & editing. LZ: Writing – review & editing. EL: Writing – review & editing. EH: Writing – review & editing. ST: Writing – review & editing. BB: Writing – review & editing. DS: Writing – review & editing. SU: Writing – review & editing.
